# Supplementary material for: Magnetic-Responsive Bendable Nozzles for Open Surface Droplet Manipulation
Source: Polymers (Basel). 2019 Nov 1;11(11):1792. doi: 10.3390/polym11111792 (PMC6918237; doi:10.3390/polym11111792)
Supplement: Supplementary file 1 [file polymers-11-01792-s001.zip › Supporting Information_Magnetic-responsive bendable nozzles_polymers_proofread.docx]

Magnetic-responsive bendable nozzles for open surface droplet manipulation

Lizbeth O. Prieto-López*, Jiajia Xu^†^, Jiaxi Cui*

INM- Leibniz Institute for New Materials, Campus D2 2 66125 Saarbrücken, Germany

KEYWORDS: droplet manipulation, magnetic responsive elastomer, open-surface microfluidics.

**Supporting Information**

**Mechanical properties of elastomeric composites**

We measured the mechanical properties of the magnetic composites with a mechanical tester Zwick 1446, Zwick/Roell, Germany. For these test we prepared beams with dimensions of 40 mm x 10 mm x 1 mm. The crosslinker and magnetic particle contents of the tested specimens are summarized in Table S1.

Table S1. Crosslinker and magnetic particle content in the elastomeric composites

| **Base to catalyst ratio** | **Crosslinker % (weight)** | **Magnetic particle % (weight)** |
| --- | --- | --- |
| 10 : 1 | 9.1 | 0, 20, 80 |
| 10 : 0.8 | 7.4 | 0, 20, 80 |
| 10 : 0.6 | 5.7 | 0, 20, 80 |
| 10 : 0.5 | 4.8 | 0, 20, 40, 60, 80 |
| 10 : 0.4 | 3.9 | 0, 80 |

The Young’s modulus was determined from tensile tests using the slope of the strain-stress curve within the region of 10 % strain. The results for the magnetic composites described in Table S1 with different combinations of crosslinker and magnetic particle content are plotted in Figure S1. We observed that the stiffness of the composite increases with both the magnetic particle content and the crosslinker content. However, the crosslinker content has a stronger influence on the stiffness of the composite than the content of magnetic particles. Using a crosslinker ratio of 10:0.5, lower than the standard 10:1, allowed us to increase the content of magnetic particles while keeping the stiffness of the composite sufficiently low for an easy bending. A Young’s modulus of ~ 1 MPa rendered enough flexibility with the aspect ratio of L/D=5 corresponding to the geometry of our nozzles. A lower *E* would produce extremely soft structures that would bundle up after demolding due to the magnetic attraction between the nozzles tips. Therefore in order to have stable structures the stiffness of the composite has to render sufficient mechanical strength of the structures to overcome the magnetic attraction between the tips.

**Figure S1.** Young’s modulus of elastomeric composites with different crosslinker and magnetic particle content.

To select an adequate content of magnetic particles the criterion was the maximum distance between a nozzle structure and a permanent magnet (neodymium magnet 1mm x 1 mm, Magnet-shop SM-01x01-N, grade N45, BH_max_ 342-358 kJ/m^3^, B_r_ = 1.32 - 1.37 T, H_c_= 860-995 kA/m, pull force ~ 25 g) to snap into contact. Figure S2 shows the maximum distance between initial position of pillars with different magnetic content and the permanent magnet before they snapped into contact. These pillars had similar geometry than the nozzles (4 mm x 800 µm) and the crossliker content was the same in all cases of 4.8 %. The permanent magnet approached with a micrometric stage from the left in steps of ca. 100 µm allowing few seconds between approaching steps to account for the viscoelastic response of the material. As expected we observed that for a given crosslinker ratio the magnetic particle content improves the response of the pillars. However this improvement of about 360 µm from 40 % to 60 % magnetic content is not significant for this application.

We evaluated the effect of the orientation of the magnetic particles within the nozzles on their bending response. We introduced the orientation of the magnetic particles relative to the principal axis of the nozzles by curing the magnetic composite under the action of an external magnetic field. Three options were evaluated: magnetic field lines along the principal axils of the nozzles labelled as ‘parallel’ (Figure S2 a and d), magnetic field lines perpendicular to the principal axils of the nozzles labelled as ‘perpendicular’ (Figure S2 b and e) and a random orientation (Figure S2 c and f) which was cured without the presence of an external field. We observed that, for a determined crosslinker and magnetic particles contents, the optimized response can be achieved with proper distribution of the magnetic particles. This corresponds to the case when the particles are randomly oriented but homogeneously distributed within the tip of the nozzles. The second best scenario is when the particles are arranged in the field lines perpendicular to the principal axis of the nozzles. The worst response corresponds to the case when the particles are arranged in the field lines along the principal axis. This is due to the structural reinforcing provided by the chain of particles formed along the principal axis which render a higher stiffness of the structure and thus higher opposition against bending. This effect has been documented before[^1-4^](#_ENREF_1).

**Figure S2.** **Orientation of magnetic particles in the nozzles.** (**a, d, g**) Parallel to principal axis (**b, e, h**) perpendicular to principal axis and (**c, f, g**) random orientation of the magnetic particles following the field lines of the external field applied during curing of the polymer.

**The nozzle surface**

In order to demonstrate the complete encapsulation of magnetic particles by PDMS, X-ray elemental analysis (EDX) of the nozzles was performed. The results show that although some particles appear to be exposed to the outermost surface of the nozzles, the particles are actually covered by a silicone layer.

**Figure S3.** **Outermost surface of the nozzles.** (**a, b, c**) Electron backscattered images of the top surface of the nozzles at different scale. (**d**) Magnetic particle aggregate on the outermost surface. (**e**) EDX analysis on the magnetic particle marked in (**d**) with a yellow cross.

The introduction of particles within the PDMS matrix did not change the chemistry of the outermost surface but it did change its morphology, as indicated by the micrographs above. This was also observed by white light interferometry; the images of PDMS and the magnetic composite revealed an increased roughness in the composite. A root-mean-squared (rms) value of ~ 7 nm was observed from PDMS surface crosslinked open to air and an rms of ~ 90 nm was observed from the composite surface (40 % magnetic particles) crosslinked open to air.

**Figure S4.** **White light interferometry images of PDMS and composite.** (**a**) PDMS surface (**b**) composite (40% magnetic particles) crosslinked open to air.

**Mixing indices at various nominal flowrates**

We carried out mixing experiments at different nominal flowrates. The image sequences and mixing indices (*MI*) plot obtained from these experiments are shown in Figure S5 for the low viscosity liquid-pair. These *MI* represent the average values from four tests of each experimental setting. The different colors in this graph correspond to different nominal flowrates in enhanced-mix configuration and the black color to sessile-mix configuration. The symbols represent the experimental data and the lines the best fit of *MI = A + B e ^Ct^* to each data set, included as guidelines. According to these results the enhanced-mix requires an average period of 30 s to achieve 80 % of the mixing and the full mixing, i.e. *MI* = 1, is reached after 50 s. This time is nearly doubled in the sessile-mix configuration which requires ~50 s to achieve 80 % of mixing and about 80 s for a complete mixing.

The results in Figure S5 show that the enhanced-mix is in general faster than the sessile-mix. Contrasting with our expectation, the increase of three orders of magnitude in the flowrate does not appear to have any effect on the mixing rate; all curves from enhanced-mix experiments practically overlap. We attribute this to the fact that the actual dispensed flowrate in our setup is similar (50 µl/min) for all nominal flowrates set between 50 µl/min to 10 ml/min at this particular small dose; as demonstrated in the next section. The faster mixing achieved at the actual dispensing flowrate of ~50 µl/min compared to that observed in sessile-mix (no chaotic advection from feeding streams available for mixing) demonstrates that the velocity from the feeding streams can enhance the mixing process and so we can expect even higher improvement for actually higher dispensing flowrates.

**Figure S5.** **Mixing of low viscosity liquid-pair**. LIF image sequence of (**a**) sessile-mix configuration and enhanced-mix configuration at nominal flowrates of: (**b**) 50 µl/min, (**c**) 2 ml/min and (**d**) 10 ml/min. (**f**) *MI* change in time for these mixing experiments.

These results of the experiments carried out with the liquid-pair of higher viscosity are shown in Fig. S6. In this case the time required for the full mixing is longer than in previous case given that viscosity not only slows down diffusive but also convective processes. However a remarkable reduction of more than 80 % in the time required for complete mixing is attained when using the enhanced-mix configuration, compared to the time required in the sessile-mix. This means a reduction from more than 8 minutes down to ~80 seconds for a full mix. The *MI* values from these experiments are plotted in Figure S6e. We also notice in the sessile-mix an initial period of about 6 s when no mixing occurs. This is also observed in the image sequence in Fig. S6a; a clear boundary between fluids is still observed 4 s after coalescence which indicates that although both fluids redistribute inside the drop no intermixing occurs. The enhanced-mix is clearly much faster than sessile-mix and similarly as before no clear effect at nominally different flowrates was observed in the enhanced-mix configuration.

**Figure S6.** **Mixing of higher viscosity liquid-pair**. LIF image sequence of (**a**) sessile-mix and enhanced-mix at nominal flowrates of (**b**) 0.5 ml/min, (**c**) 5 ml/min and (**d**) 10 ml/min. (**e**) *MI* change in time.

**The dispensing flowrate**

The true dispensing flowrate at the nozzles was carefully evaluated by measuring the diameter of drops growing at nominally different flowrate settings. The droplet diameter was measured from image sequences similar to those in Figure S7 (a) and (b); these sequences show two drops growing on the tip of a nozzle at a different nominal flowrate. The average droplet diameter is plotted against time in Figure S7(c) for different nominal flowrates. Each dataset represents the average and standard deviation from four tests. The solid gray lines denote the theoretical diameter growth (considering a spherical volume growing at the given flowrate) at the lowest and highest evaluated nominal flowrates. The dashed black line at *d*= 1.58 mm indicates the diameter of a spherical drop of 2 µl. From these results we observe that there is no clear difference between the droplet growth rates from the different nominal flowrates. Furthermore, at this particular small dose all experimental curves from nominally different flowrates are effectively described by the theoretical curve corresponding to a flowrate of 50 µl/min. According to these results the accuracy in the delivered volume of 2 µl was of ± 0.33 µl which was fully dispensed after 6 s although about 70% of the volume was supplied within the first 2 s in all settings. These measurements revealed that the delivery of fluid was actually neither very precise nor accurate at these small doses in our system. Improvement in the dispensing mechanism (see methods) is required in order to provide the true flowrates. This limited our study on the effect of increasing flowrate on the mixing time and we can only demonstrate the enhancement of the mixing at one actual dispensing flowrate of ~ 50 µl/min.

**Figure S7.** **Drops growing at various nominal flowrates settings**. LIF image sequence of water + Rhodamine drop growing on the tip of a bendable nozzle at a nominal flowrate of (**a**) 50 µl/min and (**b**) 10 ml/min. (**c**) Drop diameter growth at different nominal flowrates; the symbols represent values measured from images and different color corresponds to a different nominal flowrate. The solid gray lines represent the theoretical curves at the lowest and highest flowrates.

**Recirculation flow in drop due to feeding streams**

To demonstrate the induced recirculation flow inside a drop mixed in enhanced-mix configuration we made a test using carbon black powder which in the presence of water agglomerates in larger clusters easier to track optically. To record the movement of isolated clusters inside the water drop we used a high speed camera and used the tracking plugin from Fiji software to extract the velocity of single clusters. The image in Figure S8 shows the trajectory of one carbon powder cluster after 10 s of the initial fluid release, i.e. full volume already dispensed, at a nominal rate of 500 µl/min. The approximated average velocity was of 560 µm/s which is comparable to the velocity of 400 µm/s estimated from the nominal flow of 50 µl/min and the cross-section of a 2 µl drop. It is worth mentioning that this velocity measurement is an under estimation of the real speed given the 2D nature of the measurement of the 3D displacement of the cluster within the water drop. The convection inside the mixed drop promoted by the feeding streams is expected to be more intense and chaotic at higher flowrates which would result in a faster mixing.


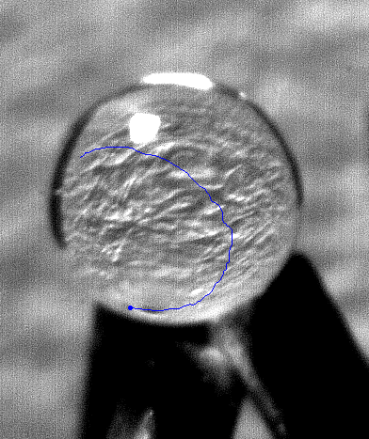


**Figure S8**. Image tracking of a carbon black cluster in a water drop mixed with enhanced-mix configuration.

**Video1:** two-step reaction

**Video2:** simultaneous mixing after splitting of yellow drop

**References**

1 Varga, Z., Fiipcsei, G. & Zrinyi, M. Magnetic field sensitive functional elastomers with tuneable elastic modulus. *Polymer* **47**, 227-233, doi:10.1016/j.polymer.2005.10.139 (2006).

2 Krommenhoek, P. J. & Tracy, J. B. Magnetic Field-Directed Self-Assembly of Magnetic Nanoparticle Chains in Bulk Polymers. *Particle & Particle Systems Characterization* **30**, 759-763, doi:10.1016/j.polymer.2005.10.139 (2013).

3 Drotlef, D.-M., Blümler, P., Papadopoulos, P. & del Campo, A. Magnetically Actuated Micropatterns for Switchable Wettability. *ACS Applied Materials and Interfaces* **6**, 8702-8707, doi:dx.doi.org/10.1021/am5014776 (2014).

4 Drotlef, D.-M., Blümler, P. & Del Campo, A. Magnetically actuated patterns for bioinspired reversible adhesion (dry and wet). *Advanced Materials* **26**, 775-779, doi:10.1002/adma.201303087 (2014).
